# Effects of a Diet Supplemented with Exogenous Catalase from *Penicillium notatum* on Intestinal Development and Microbiota in Weaned Piglets

**DOI:** 10.3390/microorganisms8030391

**Published:** 2020-03-11

**Authors:** Yang Li, Xilun Zhao, Lijia Zhang, Xiaoyan Zhan, Zhiguo Liu, Yong Zhuo, Yan Lin, Zhengfeng Fang, Lianqiang Che, Bin Feng, Shengyu Xu, Jian Li, De Wu

**Affiliations:** 1 Key Laboratory for Animal Disease-Resistance Nutrition of the Ministry of Agriculture, Animal Nutrition Institute, Sichuan Agricultural University, Huimin Road 211#, Chengdu 611130, China; liyang_cc@yeah.net (Y.L.);; 2 Department of Animal Science and Technology, Shanghai Vocational College of Agriculture and Forestry, Zhongshan Second Road 658#, Shanghai 201699, China; 3 Liaoning Vetland Bio-Technology Co., Ltd., Lingdong Street, Shenyang 110033, China

**Keywords:** weaned piglets, catalase, intestine, microbiota, health

## Abstract

This study aims to investigate the effects of exogenous catalase (CAT), an antioxidative enzyme from microbial cultures, on intestinal development and microbiota in weaned piglets. Seventy-two weaned piglets were allotted to two groups and fed a basal diet or a basal diet containing 2.0 g/kg exogenous CAT. Results showed that exogenous CAT increased (*p* < 0.05) jejunal villus height/crypt depth ratio and intestinal factors (diamine oxidase and transforming growth factor-α) concentration. Moreover, dietary CAT supplementation enhanced the antioxidative capacity, and decreased the concentration of pro-inflammatory cytokine in the jejunum mucosa. Exogenous CAT did not affect the concentration of short-chain fatty acids, but decreased the pH value in colonic digesta (*p* < 0.05). Interestingly, the relative abundance of *Bifidobacterium* and *Dialister* were increased (*p* < 0.05), while *Streptococcus* and *Escherichia-Shigella* were decreased (*p* < 0.05) in colonic digesta by exogenous CAT. Accordingly, decreased (*p* < 0.05) predicted functions related to aerobic respiration were observed in the piglets fed the CAT diet. Our study suggests a synergic response of intestinal development and microbiota to the exogenous CAT, and provides support for the application of CAT purified from microbial cultures in the feed industry.

## 1. Introduction

Healthy intestines are not only beneficial to the digestion and absorption of nutrients, but also a crucial safeguard against the invasion of pathogens [1]. Gut microbiota is a category of microorganisms inhabiting the mammalian gastrointestinal tract, and are involved in maintaining intestinal health in human and monogastric animals, including providing nutrients, optimizing the intestinal immune system, and regulating the growth of the intestinal epithelial mucosa [2,3]. Weaning a pig from its sow is one of the most stressful events in a pig’s life [4]. Weaned piglets suffer from a huge stress response (especially oxidative stress) and changes in gut microbiota because of multiple factors such as environmental factors, weaning, and infection [5,6]. A previous study demonstrated that oxidative stress is strongly linked to the alteration of gut microbiota [7]. Moreover, oxidative stress suppresses intestinal development [8], weakens intestinal digestion and absorption [9], and induces mucosa inflammation and cell apoptosis [10], which can result in growth restriction, disease, and even death in piglets [11,12]. Therefore, decreasing postweaning oxidative stress is crucial for weaned piglets.

Hydrogen peroxide (H_2_O_2_), belonging to reactive oxygen species (ROS), is constantly generated from oxygen in all aerobic metabolism and pathogenic processes [13]. Excessive H_2_O_2_ is harmful to almost all cell components, so its rapid and efficient removal is essentially important for aerobically living organisms [14]. Catalase (CAT), an important antioxidative enzyme in the body, can break H_2_O_2_ down into O_2_ and H_2_O and reduce its damage to the body [15]. In turn, excess ROS also inhibits catalase activity [16]. Currently, exogenous CAT, which is purified from animal tissues or microorganisms [17,18], has been used in pharmaceuticals [19] and the food industry [20], as well as by biocatalyst [21] and other industries [22]. Wang [23] suggested that exogenous CAT supplementation in high-fat diets could increase rat antioxidative capacity and improve the intestinal flora structure. Our recent study showed that piglets fed diets supplemented with exogenous CAT had higher gain-to-feed ratios [24]. However, there was little information available in the scientific literature on the evaluation of the effects of dietary supplementation with exogenous CAT on intestinal development and gut microbial composition of weaned piglets.

In the present study, we investigated the effects of dietary exogenous CAT from microbial culture supplementation on the intestinal development and gut microbiota of weaned piglets, which could be helpful to understanding the microbial mechanisms behind exogenous CAT-modulated intestinal development, and could provide support for the application of CAT purified from microbial fermentation in the feed industry.

## 2. Materials and Methods 

### 2.1. Ethical Approval

The present experiment was conducted at the Research Farm of Animal Nutrition Institute, Sichuan Agricultural University, Ya’an, China. The research protocol was approved by the Care and Use committee of Sichuan Agricultural University under ethic approval number SCAUAC201408-3.

### 2.2. Exogenous Catalase Production

The exogenous CAT production was provided by Liaoning Vetland Bio-Technology Co., Ltd., Liaoning, China. It was produced from *Penicillium notatum* (collection number: ACCC 30443), followed by spray drying and sieving to obtain powder production.

### 2.3. Experimental Design and Animal Management

A total of 72 Duroc × (Landrace × Yorkshire) crossbred weaned piglets (21 d of age, 6.90 ± 0.01 kg) were selected as experimental animals and housed in 12 pens with 6 piglets per pen. Pens were randomly assigned to one of the following two dietary treatments (*n* = 6) for a 35-d feed trial: (1) CON group (piglets were fed with a basal diet) and (2) CAT group (piglets were fed with the basal diet supplemented with 2.0 g/kg exogenous CAT). Diets were offered to piglets according to two-phase feeding programs (1–21 d and 22–35 d of trial). The basal diets were formulated to respect or exceed the nutrient requirements recommended by the National Research Council (NRC, 2012) for weaned piglets at different growth stages and the ingredient composition and nutrient levels are shown in Supplementary Table S1. The exogenous CAT was added to the basal diets at the expense of corn. Piglets were housed in a totally enclosed and temperature-controlled room with 12 pens (1.5 × 2.5 m) and had free access to feed and water [25]. The room temperature was set at 28 ± 1 °C for the first week and gradually decreased to 25 °C by the end of the trial. No vaccines or antibiotics were administered to these piglets throughout the experiment.

### 2.4. Sampling Procedure

At the end of the feeding trial, one healthy pig per pen with a similar body weight (BW) to the average pen weight (totally 12 piglets, 14.62 ± 0.20 kg) was selected and slaughtered immediately after deep anesthesia with Zoletile 50 (Virbac, France) administered by intramuscular injection at a dose of 0.1 mg/kg of BW. After evisceration, the small intestine was dissected and rapidly measured for weight. The small intestine in piglets was defined as described in Li et al. [26]. Briefly, intestinal segments (duodenum, jejunum, and ileum) were obtained by using the anatomical landmarks, with the pyloric-duodenal junction to the duodenal-jejunal junction being the duodenum, the duodenal-jejunal junction to the jejunal-ileal junction being the jejunum, and the jejunal-ileal junction to the ileocecal junction being the ileum. The relative weight of the intestine was calculated as the intestinal weight divided by the BW of piglet. The intestinal segments (about 2 cm) of tissues (duodenum, jejunum, and ileum), were immediately rinsed by physiological saline and fixed in 4% paraformaldehyde for 24 h for morphological examination. The rest jejunum was opened longitudinally and washed in ice-cold physiological saline solution. The mucosa tissue from jejunum was subsequently collected by scraping using a sterile glass slide, followed by being snap-frozen in liquid nitrogen and being stored at −80 °C until further analysis. The colonic digesta samples were collected on a clean bench, immediately placed in sterile bags, and stored at −80 °C for further analysis.

### 2.5. Mucosal Morphology Measurement

Fixed intestinal segments were embedded according to routine paraffin-embedding protocol. Serial sections with a 5-μm thickness were made using a microtome, and this was followed by hematoxylin and eosin staining. Villi height and crypt depth were determined as previously described in Li et al. [27]. Briefly, two transverse sections of each intestinal sample were prepared on one slide for morphometric analysis. A total of 12–20 intact, well-oriented crypt-villus units per sample were selected randomly and measured. Villus height measurements were taken from the tip to the base of the villus between individual villi, and crypt depth was measured from the valley between individual villi to the basal membrane. The crypt depth (μm) and villus height (μm) of the small intestine were measured using an Olympus BX51 microscope equipped with a DP70 digital camera (Olympus, Tokyo, Japan) and JD801 morphologic image analysis software (JEDA, Nanjing, Jiangsu, China). The ratio of villus height to crypt depth (VCR) was calculated as the villus height divided by the crypt depth.

### 2.6. Determination of H_2_O_2_ in Intestinal Tissue

The H_2_O_2_ level in jejunal mucosa tissues was determined according to the manufacturer’s instruction (Beyotime Biotech, Shanghai, China) as previously described in Luo et al. [5]. Briefly, the mucosa tissues were weighted and homogenized in an H_2_O_2_ lysis buffer (1:20, *w*/*v*) followed by centrifugation at 12,000× *g* for 10 min for supernatants. The absorbance was read at 560 nm after the sample solution (50 µL) was incubated with reaction solution (100 µL) at room temperature for 30 min. The H_2_O_2_ concentration was calculated by the standard curve made from the standard solutions.

### 2.7. Intestinal Antioxidant Parameters Determination

The determination of intestinal antioxidant parameters was proceeded as previously described in Chen et al. [8]. The jejunal mucosa samples were homogenized in ice-cold saline solution (1:9, *w*/*v*), followed by centrifugation at 2500× *g* for 10 min at 4 °C. The supernatant was prepared for further determination. Intestinal mucosa antioxidant parameters included CAT, total antioxidant capacity (T-AOC), superoxide dismutase (SOD), glutathione peroxidase (GSH-Px), and malondialdehyde (MDA), and were measured with the commercial kits (Nanjing Jiancheng Institute of Bioengineering, Nanjing, Jiangsu, China) according to the manufacturer’s instructions.

### 2.8. Intestinal Barrier Integrity Determination

The determination of jejunal mucosal barrier integrity was proceeded as described in a previous study [28]. Diamine oxidase (DAO) activities, transforming growth factor-α (TGF-α), trefoil factor family (TFF), and major histocompatibility complex class II (MHC-II) were analyzed by commercial porcine-specific ELISA kits (Beijing winter song Boye Biotechnology Co. Ltd., Beijing, China) according to the manufacturer’s instructions.

### 2.9. Intestine Mucosal Proinflammatory Cytokine and SIgA Determination

Jejunal mucosa was homogenized in ice-cold physiological saline (1:9, *w*/*v*) and the supernatant was collected. The levels of tumor necrosis factor-α (TNF-α) and interleukin-6 (IL-6) in the jejunal mucosa were measured using a commercially available ELISA kit (R&D Systems Inc., Minneapolis, MN). The concentration of secretory immunoglobulin A (SIgA) in jejunum was determined using the sandwich ELISA kits (Beijing winter song Boye Biotechnology Co. Ltd., Beijing, China) according to the manufacturer’s instructions.

### 2.10. Digesta pH Values Determination

The method of digesta pH value determination was according to Chen et al. [28]. Briefly, about 5 g colonic digesta of each pig was immediately transferred into an ice-bathed sterile 10-mL centrifugal tube after the colon segments were removed, and the pH value of each sample was measured by pH meter (PHS-3C PH, Shanghai, China).

### 2.11. Short-Chain Fatty Acids Concentration Analysis

The short-chain fatty acids (SCFAs) in piglet colonic digesta were measured according to the protocol from a previous study [29]. Briefly, 0.7 g of colonic digesta samples were suspended in 1.5 mL of distilled water and allowed to stand for 30 min, followed by being centrifuged for 15 min at 15,000× *g* at 4 °C. Then 1 mL supernatant was transferred and mixed with metaphosphoric acid (0.2 mL, 25%, *w*/*v*) and crotonic acid (23.3 µL, 210 mmol/L). After standing at 4 °C for 30 min, the samples were centrifuged for 10 min at 15,000× *g* again. Then the supernatant was transferred and mixed with chromatographic methanol (1:1, *v*/*v*). After centrifugation at 10,000× *g*, an amount of 1 µL supernatant was analyzed using gas chromatography (Varian CP-3800 GC, USA).

### 2.12. Microbial Analysis

Bacterial genomic DNA was extracted from frozen colonic samples with the EZNA TM Stool DNA kit (Omega Bio-Tek, Norcross, Georgia, USA) according to the manufacturer’s protocol. DNA concentration and purity were monitored on 1% agarose gels. According to the concentration, DNA was diluted to 1 ng/μL using sterile water. The barcoded V4: 515F-806R primer (5′-GTGCCAGCMGCCGCGGTAA-3′ and 5′-GGACTACHVGGGTWTCTAAT-3′, respectively) were used to perform amplicon pyrosequencing on the Illumina HiSeq PE2500 platform (Novogene, Beijing, China). Generated 250-bp paired-end reads from the original DNA fragments were merged using Fast Length Adjustment of Short reads (FLASH) [30]. The sequences containing ambiguous bases or mismatches in the primer regions were filtered to obtain the high-quality clean sequence tags according to the Quantitative Insights Into Microbial Ecology (QIIME, V1.7.0) quality control process [31,32]. All sequencing data are available in the NCBI Sequence Read Archive (SRA) under accession PRJNA560659 (Illumina sequences). Then the tags were compared with the Gold database using the UCHIME algorithm to detect chimera sequences [33,34]. The effective tags were finally obtained after the chimera sequences were removed. The effective tags were performed by Uparse software (V7.0.1001) and sequences were clustered into the same operational taxonomic units (OTUs) at 97% sequence similarity [35], and were classified to different levels (phylum, class, order, family, genus, and species) by comparing sequences with the GreenGene database [36] using the Ribosomal Database Project (RDP) classifier (V2.2) [37]. Alpha and beta diversity were both calculated to compare the taxonomic data. The observed species (calculated unique OTUs), Shannon index (measured community diversity), and Chao 1 index (estimated community richness) were used to ascertain differences in alpha diversity based on different diets. A Wilcoxon rank-sum test was used to detect the statistical differences between two groups. Bray–Curtis distances were calculated and visualized by Principal Coordinate Analysis (PCoA) [38]. An analysis of similarity (ANOSIM) test was used to access significant differences among the microbial communities. All the analyses from clustering to alpha and beta diversity were performed with QIIME (V1.7.0) and displayed with R software (V2.15.3). Functional profiles of the prokaryotic community were annotated using the Functional Annotation of Prokaryotic Taxa (FAPROTAX) [39].

### 2.13. Statistical Analysis

The individual piglet was considered to be the experimental unit for all other variables. All indexes except microbial analysis were used to evaluate significance using the t-test procedure of SAS 9.0 (Institute Inc., Cary, NC, USA). Normality of the data was assessed using a Shapiro–Wilk’s statistic (W > 0.05). If the data did not follow a normal distribution, transformation was used to achieve normality. Values are expressed as mean ± standard error in tables and figures. Statistical significance was set at *p* < 0.05, and 0.05 < *p* < 0.10 was considered a trend toward significance.

## 3. Results

### 3.1. Effects of Exogenous CAT on Intestinal Relative Weight and Morphology

Effect of dietary exogenous catalase supplementation on the intestinal relative weight of weaned piglets is presented in Table 1. Piglets fed diets supplemented with exogenous CAT had heavier (*p* < 0.05) ileums and small intestines, and had a trend of increased jejunum weight compared with those fed the CON diet (*p* < 0.10).

As shown in Table 2, the jejunal villus height and VCR of piglets fed the CAT diet were significantly higher than of those fed the CON diet (*p* < 0.05), and the duodenal villus height of piglets in the CAT group tended to be higher than of those in the CON group (*p* = 0.051). No significant difference was observed in ileum indexes (*p* > 0.05).

### 3.2. Effects of Exogenous CAT on Intestinal Antioxidative Capacity

Effects of dietary exogenous catalase supplementation on the jejunum mucosa antioxidant parameters of weaned piglets are shown in Table 3. The jejunal mucosa CAT and SOD activity of piglets in the CAT group were significantly higher than in the CON group (*p* = 0.040), and piglets fed the CAT diet had significantly lower concentrations of MDA and H_2_O_2_ (*p* < 0.05). No significances were observed in GSH-Px and T-AOC activities (*p* > 0.05).

### 3.3. Effects of Exogenous CAT on Intestinal Mucosa Barrier Function

The concentrations of intestinal factors, DAO, and TGF-α in the jejunal mucosa of piglets fed the CAT diet were both significantly higher than piglets fed the CON diet (*p* < 0.05, Table 4). The concentrations of TFF and MHC-II were not significantly affected by dietary treatment (*p* > 0.05).

### 3.4. Effects of Exogenous CAT on Intestinal Mucosa Proinflammatory Cytokines and SIgA Concentration

Effects of dietary exogenous catalase supplementation on the jejunum mucosal proinflammatory cytokine and SIgA of weaned piglets are shown in Table 5. Dietary CAT significantly decreased (*p* < 0.05) the level of TNF-α and IL-6, and significantly increased (*p* = 0.025) the concentration of SIgA in the jejunum mucosa.

### 3.5. Effects of Exogenous CAT on Concentration of SCFAs and pH Values in Colonic Digesta

As shown in Table 6, significantly lower pH values were observed in the colonic digesta of piglets fed the CAT diet compared with those fed the CON diet (*p* = 0.006). Piglets fed the CAT diet tended to have a higher butyrate concentration in colonic digesta (*p* = 0.074).

### 3.6. Effects of Exogenous CAT on Microbial Diversity in Colonic Digesta

**Figure 1:**
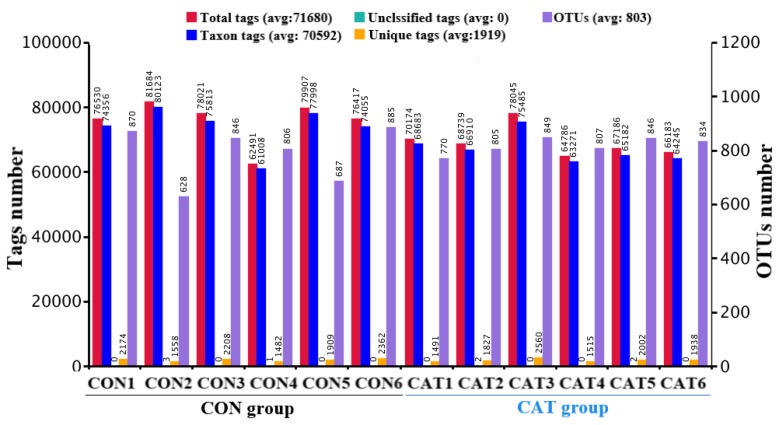
Operational taxonomic unit (OTU) clustering and annotation per sample. CON 1, 2, 3, 4, 5, and 6 are colonic digesta samples from piglets fed with a basal diet; CAT 1, 2, 3, 4, 5, and 6 are colonic digesta samples from piglets fed with a basal diet supplemented with 2.0 g/kg exogenous catalase production.

Total tags, taxon tags, unclassified tags, unique tags, and OTUs are shown in Figure 1. A total of 847,129 taxon tags were obtained from two treatments, with an average of 70,594.08 ± 1857.12 per sample. The species diversity of the samples was further studied and species were annotated on the representative sequence of OTUs. A total of 4722 OTUs were found in the CON group, with an average of 787.00 ± 43.06 per sample, while a total of 4911 OTUS were found in the CAT group, with an average of 818.50 ± 12.37 per sample.

**Figure 2:**
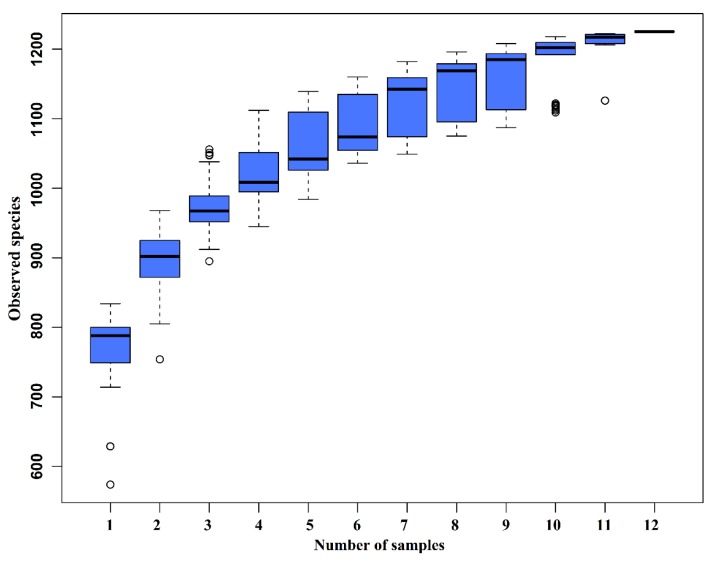
Species accumulation curves (SAC). The SAC tends to flatten as the number of analyzed sequences increases up to 12, indicating that our samples were sufficient for OTU testing and could predict the species richness of samples.

The species accumulation curves (SAC) (Figure 2) tended to flatten as the number of analyzed sequences increased up to 12, indicating that our samples were sufficient for OTU testing and could predict the species richness of samples.

**Figure 3:**
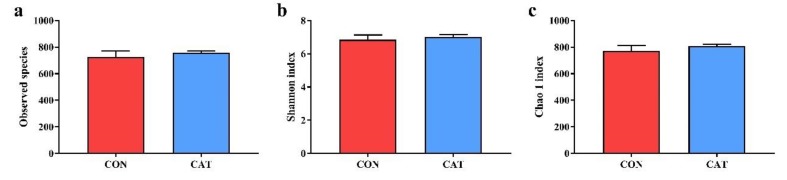
Difference on bacteria community diversity and richness between the two treatments. (**a**) Observed species; (**b**) Shannon index; (**c**) Chao 1 index. CON, piglets fed with a basal diet; CAT, piglets fed with a basal diet supplemented with 2.0 g/kg exogenous catalase production. Values are mean ± standard error (*n* = 6).

To assess colonic microbial community structures, observed species (Figure 3a), Shannon index (Figure 3b), and Chao 1 index (Figure 3c) were calculated, but no significant differences were observed in the three indexes (*p* > 0.05).

**Figure 4:**
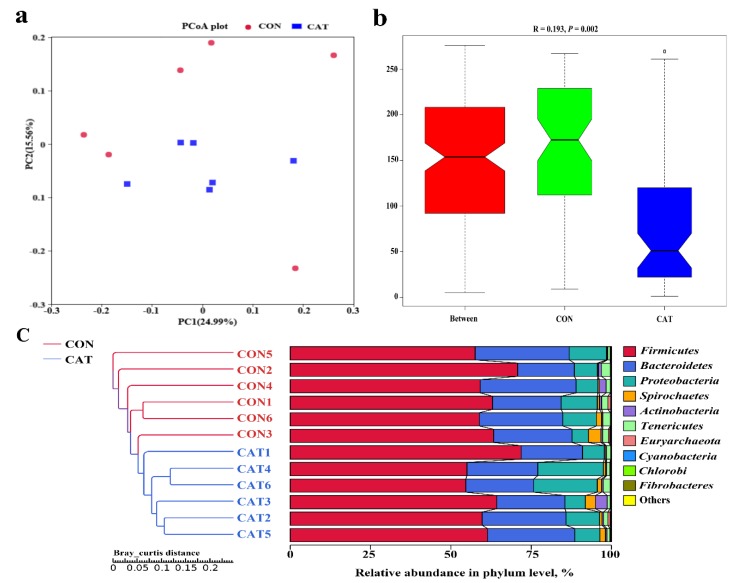
The beta diversity of microbial communities in two treatments. (**a**) The principal coordinate analysis (PCoA) profile of the two groups displayed with the Bray–Curtis distance. Each dot represents one sample from each group. The percent variation explained by each principal coordinate is indicated on the X and Y axis. (**b**) Analysis of similarity (ANOSIM). R value is scaled to lie between −1 and +l. Generally, 0 < R < 1 and *p* < 0.05 represents that there were significant differences between the groups. (**c**) Unweighted pair-group method with arithmetic mean (UPGMA) phylogenetic tree constructed based on the Bray–Curtis distance. The left panel shows the phylogenic tree, and the right panel shows the relative abundance of each sample at the phylum level. CON 1, 2, 3, 4, 5, and 6 are colonic digesta samples from piglets fed with a basal diet; CAT 1, 2, 3, 4, 5, and 6 are colonic digesta samples from piglets fed with a basal diet supplemented with 2.0 g/kg exogenous catalase production.

The PCoA profile for piglet colonic samples based on the Bray–Curtis distance (Figure 4a) showed a clear separation between the CON group and CAT group. The ANOSIM (Figure 4b) showed that two groups had significantly different microbiota community structures (*p* < 0.05). An unweighted pair-group method with an arithmetic mean (UPGMA) phylogenetic tree (Figure 4c) constructed based on the Bray–Curtis distance showed that samples CON1, CON2, CON3, CON4, CON5, and CON6 formed the first group, while CAT1, CAT2, CAT3, CAT4, CAT5, and CAT6 formed the second group, suggesting the two groups had obvious differences in their microbial communities.

### 3.7. Effects of Exogenous CAT on Microbial Relative Abundances in Colonic Digesta

The relative abundance at the phylum level in piglet colonic microbiota (top 10 phyla) is shown in Figure 4c. *Firmicutes* and *Bacteroidetes* were the most predominant phyla. There was no significant difference observed in the top 10 phyla between the CON group and the CAT group (*p* > 0.05).

**Figure 5:**
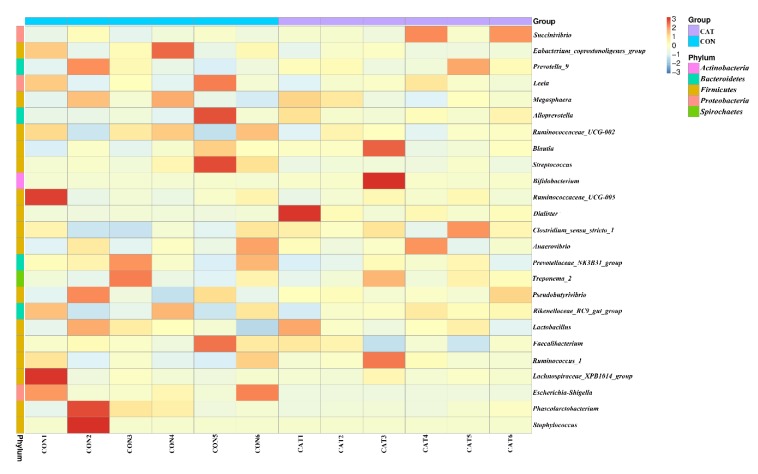
Bacterial community heatmap analysis at the genus level. CON 1, 2, 3, 4, 5, and 6 are colonic digesta samples from piglets fed with a basal diet; CAT 1, 2, 3, 4, 5, and 6 are colonic digesta samples from piglets fed with a basal diet supplemented with 2.0 g/kg exogenous catalase production.

The relative abundance at the genus level in piglet colonic microbiota (top 25 genera) is shown in Supplemental Table S2. The colonic samples were dominated by three genera: *Lactobacillus*, *Succinivibrio*, and *Alloprevotella*. The heatmap (Figure 5) exhibits the abundance of the selected genera across all the samples, clearly showing that there were significant differences in the genus distribution between the CON and CAT treatments. Dietary exogenous CAT supplementation significantly decreased the relative abundance of *Streptococcus* (*p* = 0.021) and *Escherichia-Shigella* (*p* = 0.006), and significantly increased the relative abundance of *Dialister* (*p* = 0.008) and *Bifidobacterium* (*p* = 0.023).

### 3.8. Effects of Exogenous CAT on Microbial Functional Characteristics

**Figure 6:**
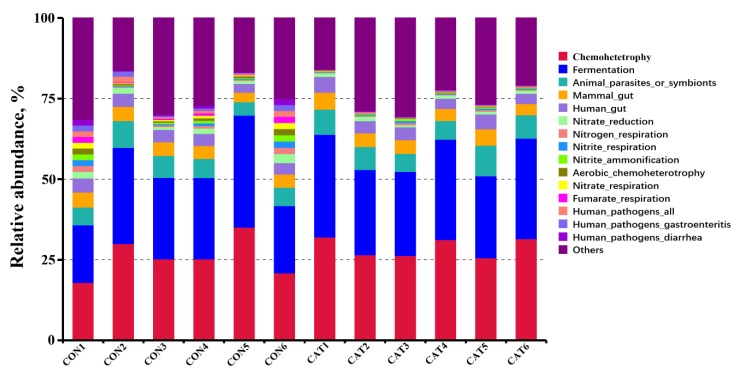
Relative abundance of the predicted function of colonic microbiota in two experimental treatments. CON 1, 2, 3, 4, 5, and 6 are colonic digesta samples from piglets fed with a basal diet; CAT 1, 2, 3, 4, 5, and 6 are colonic digesta samples from piglets fed with a basal diet supplemented with 2.0 g/kg exogenous catalase production.

Relative abundance of the top 10 predicted functions of colonic microbiota in two experimental treatments are shown Supplemental Table S3 and displayed with a bar chart in Figure 6. Microbial functions were predicted using FAPROTAX based on the relative abundance of colonic microbes. Of the 10 functions, “Chemoheterotrophy” and “Fermentation” were the top two functional annotations in the two treatments. Dietary exogenous CAT supplementation significantly decreased (*p* < 0.05) the relative abundance of “Aerobic_chemoheterotrophay”, “Nitrate_respiration”, “Fumarate_respiration”, “Human_pathogens_all”, “Human_pathogens_gastroenteritis”, and “Human_pathogens_diarrhea”, and tended to decrease (*p* < 0.10) the relative abundance of “Nitrate_reduction”, “Nitrogen_respiration”, and “Nitrite_respiration”.

## 4. Discussion

In the current study, dietary CAT supplementation increased the weight of ileums and small intestines, and benefited jejunal villus height and VCR. The weight of intestines has been considered to be an important indicator of intestine development [26], and it has been thought that higher intestinal weight is related to a thicker intestinal wall and mucus layer [40,41]. Intestinal morphology is an important indicator of the health of intestines. The height of the villus determines the ability of the small intestine to absorb nutrients, and the VCR is regarded as a reliable measure to evaluate nutrient absorption capacity of the small intestine [11]. Decreased villus height and VCR are always accompanied by a decrease in digestibility [42]. It was illuminated in this study that exogenous CAT might be conducive to digestion and the absorption of nutrients in the small intestine. Consistently, a previous study showed that exogenous supplementation increased feed conversion efficiency [24]. Further, the maturity and integrity of the intestinal epithelium barrier, which plays a key role in the digestion and absorption of nutrients and provides a physical barrier to protect against infection in the intestinal tract, were evaluated through measuring the concentration of DAO, TGF-α, TFF, and MHC-II in the jejunal mucosa. In the current study, exogenous CAT supplementation increased the concentration of DAO and TGF-α. DAO was found in high concentrations in the small intestine. In the gut, DAO is synthesized by maternal enterocytes and stored in a plasma membrane-associated vesicular structure in the intestinal epithelial cells, and is usually considered to be an indicator by which to evaluate the integrity of the intestinal barrier [43,44]. TGF-α, which serves as a polypeptide ligand of the epidermal growth factor or TNF-α receptor, has been reported as being produced by the intestinal epithelial cell and able to maintain the integrity of intestinal epithelial cells [45]. A previous study showed that increased DAO and TGF-α concentrations were associated with enhanced antioxidative capacity in the jejunal tissue [11,28]. The results also suggested a beneficial effect of exogenous CAT on intestinal epithelial barrier functions.

Intestine mucosal damage was usually associated with inflammation [8]. Previous studies have reported that weaning stress could easily trigger intestinal inflammation in pigs [46]. Dietary exogenous CAT supplementation decreased the concentration of pro-inflammatory factors (TNF-α and IL-6) and increased the concentration of SIgA. TNF-α is a pleiotropic cytokine produced by activated macrophages, with some metabolic effects on lipid metabolism [47]. TNF-α signaling can induce activation of the NF-κB signaling pathway, which is considered to be a key inducer of inflammation and programmed cell death [48]. TNF-α can also activate IL-6 production [49], which is in accord with the increased IL-6 level in the present study. IL-6, considered to be an inflammatory biomarker, is a multifunctional cytokine that plays a central role in regulating the balance between the IL-17-producing Th17 cells and regulatory T cells [50]. Activated Th17 cells can produce inflammatory mediators leading to chronic inflammation [51]. SIgA is secreted by intestinal lamina propria plasmacytes, and serves as the first line of specific defense in the mucosal immune system of the intestine [52]. The concentration of intestinal SIgA is reduced along with the number of bacteria adhered to the mucosa as it increases [53].

Early weaned piglets are prone to oxidative stress, which often causes damage to the intestinal mucosa [8]. A previous study showed that decreasing oxidative stress was a main reason for improving intestinal development in weaned piglets [28]. In the current study, the redox status in the jejunal mucosa was evaluated through monitoring the oxidative and antioxidative items. We found that dietary exogenous CAT decreased H_2_O_2_ and MDA concentrations in the jejunal mucosa of piglets. H_2_O_2_ is one of the major ROSs in the body, and MDA, a secondary product of lipid oxidation, has been widely considered to be an index by which to monitor the degree of lipid peroxidation [29]. Both H_2_O_2_ and MDA are closely associated with cell damage. A previous study showed that piglets weaned earlier than normal suckling piglets always had higher H_2_O_2_ and MDA levels and lower antioxidative enzymes activities in the intestinal tissues [54]. It has been suggested that piglets fed a diet supplemented with exogenous CAT have lower oxidative stress, which might be related to the increased activities of intestinal CAT and SOD. CAT and SOD are endogenous to important antioxidative enzymes, playing important roles in preventing oxidative damage. SOD can convert ROSs into H_2_O_2_, then CAT can degrade the H_2_O_2_ to water and oxygen [55]. The results in the mice also showed that diets enriched with exogenous CAT could increase intestinal antioxidative enzyme activities such as CAT, SOD, and GSH-Px, while alleviating the intestinal oxidative stress induced by a high-fat diet [23]. Therefore, exogenous CAT could improve intestinal development by enhancing intestinal antioxidative capacity.

Gut microbiota plays a vital role in host health by providing nutrients, modulating gastrointestinal development, shaping the immune system, and competitively inhibiting pathogens [56,57], while the gastrointestinal tract offers a physical environment for microorganisms [28]. The intestinal mucosa is a particularly dynamic environment where the host constantly interacts with trillions of commensal microorganisms, and periodically interacts with pathogens of diverse natures [58]. A mature mucosa barrier serves as a primary innate defense against pathogens, and an increased abundance of harmful bacteria will destroy the dynamic environment and result in intestinal barrier dysfunction [44]. Oxidative stress will modify the intestinal barrier function and finally contribute to the pathogenesis of gut inflammation and microbial flora disorders [7,10]. A previous study showed that dietary exogenous CAT supplementation could induce changes in the intestinal microflora of mice [23]. Similarly, exogenous CAT supplementation significantly changed the structures of gut microbiota in weaned piglets in the current study. Moreover, exogenous CAT increased the relative abundance of *Bifidobacterium* and *Dialister*, and decreased the relative abundance of *Streptococcus* and *Escherichia-Shigella*. *Bifidobacterium*, considered to be a probiotic bacteria, could decrease inflammation by inhibiting the growth of pathogens via the production of organic acids, and could release soluble factors that suppress the secretion of pro-inflammatory cytokines by immune cells [59,60]. A decreased pH value and harmful bacteria (*Streptococcus* and *Escherichia-Shigella*) were found in the colonic digesta of piglets fed the CAT diet in the present study. Wang et al. [61] have also reported that supplementation of *Bifidobacteria* improves gut barrier function by reducing the damage of the intestinal mucosa. Moreover, the abundance of *Bifidobacterium* was positive to antioxidative capacity [62]. Members of the *Dialister* genus are asaccharolytic obligately anaerobic gram-negative coccobacilli, and negatively associated with pro-inflammatory cytokine response [63,64]. *Streptococcus* and *Escherichia-Shigella* are both pathogenic bacteria. Higher abundance of *Streptococcus* is related to numerous inflammatory response [65], and *Escherichia-Shigella* is prevalent in patients with inflammatory bowel disease [66]. This indicates that dietary exogenous CAT supplementation increases the abundance of beneficial bacteria and decreases the abundance of harmful bacteria. The results of predicted functions are associated with changes in gut microbiota. The intestinal microflora is dominated by diverse anaerobes, providing both a health benefit to the host and a barrier to infection [67,68,69]. *Escherichia-Shigella*, a facultative anaerobe, can use nitrate as a terminal electron acceptor for anaerobic respiration [70], while *Bifidobacterium* and *Dialister* are anaerobic bacteria. It was reported that the inflammatory host response selectively enhances the growth of commensal *Enterobacteriaceae* by generating electron acceptors for anaerobic respiration [71]. In our study, decreased predicted functions related to aerobic respiration, such as “Aerobic_chemoheterotrophay”, “Nitrate_respiration”, and “Fumarate_respiration”, were observed in the piglets fed the CAT diet. The results suggest that dietary exogenous CAT could promote a proliferation of beneficial bacteria and inhibit the proliferation of harmful bacteria.

## 5. Conclusions

In conclusion, the present study indicated that a diet supplemented with exogenous CAT from *Penicillium notatum* shows beneficial effects in improving intestinal development and function, and in increasing the abundance of beneficial bacteria in the colons of weaned piglets. These findings will be helpful in enhancing our understanding of the mechanisms of dietary exogenous CAT in modulating gut health, and will provide support for the application of CAT purified from microbial cultures in the feed industry.

## Figures and Tables

**Table 1:** Effect of dietary exogenous catalase supplementation on the intestinal relative weight of weaned piglets.

Items	Treatment ^1^		*p* value
	CON	CAT	
Body weight ^2^, kg	14.62 ± 0.24	14.62 ± 0.35	1.000
Duodenum, %	0.10 ± 0.007	0.11 ± 0.009	0.488
Jejunum, %	3.75 ± 0.07	4.07 ± 0.14	0.064
Ileum, %	0.12 ± 0.008	0.17 ± 0.008	0.002
Cecum, %	0.78 ± 0.03	0.69 ± 0.09	0.344
Colon, %	2.08 ± 0.09	2.22 ± 0.30	0.663
Small intestine, %	3.97 ± 0.08	4.34 ± 0.15	0.049
Large intestinal ^3^, %	2.86 ± 0.09	2.91 ± 0.32	0.879
Total intestine ^3^, %	6.83 ± 0.12	7.25 ± 0.40	0.331

Values are mean ± standard error (*n* = 6). ^1^ CON, piglets fed basal diet; CAT, piglets fed basal diet supplemented with 2.0 g/kg exogenous catalase production. ^2^ Average body weights of piglets slaughtered in CON group and CAT group, respectively. ^3^ Rectum was not included.

**Table 2:** Effects of dietary exogenous catalase supplementation on intestinal morphology in weaned piglets.

Items	Treatment ^1^		*p* value
	CON	CAT	
Duodenum			
Villus height, μm	223.89 ± 20.46	277.70 ± 11.18	0.051
Crypt depth, μm	241.39 ± 20.58	279.93 ± 19.38	0.204
VCR ^2^	0.94 ± 0.06	1.02 ± 0.09	0.485
Jejunum			
Villus height, μm	201.46 ± 12.24	277.39 ± 15.22	0.003
Crypt depth, μm	171.26 ± 12.40	171.24 ± 15.46	0.999
VCR ^2^	1.20 ± 0.09	1.69 ± 0.18	0.035
Ileum			
Villus height, μm	211.19 ± 16.50	220.89 ± 13.34	0.657
Crypt depth, μm	160.27 ± 14.02	188.33 ± 30.68	0.425
VCR ^2^	1.40 ± 0.21	1.31 ± 0.20	0.768

Values are mean ± standard error (*n* = 6). ^1^ CON, piglets fed basal diet; CAT, piglets fed basal diet supplemented with 2.0 g/kg exogenous catalase production. ^2^ VCR, the ratio of villus height to crypt depth.

**Table 3:** Effects of dietary exogenous catalase supplementation on jejunum mucosa antioxidant parameters in weaned piglets.

Items ^2^	Treatment ^1^		*p* value
	CON	CAT	
CAT, U/mg protein	6.14 ± 0.64	8.44 ± 0.74	0.040
SOD, U/mg protein	19.88 ± 0.41	21.48 ± 0.45	0.025
GSH-Px, U/mg protein	17.63 ± 0.82	19.19 ± 0.82	0.209
T-AOC, U/mg protein	0.52 ± 0.02	0.55 ± 0.02	0.294
MDA, mmol/mg protein	1.24 ± 0.19	0.66 ± 0.13	0.032
H_2_O_2_, mmol/g	19.06 ± 1.36	13.57 ± 1.33	0.016

Values are mean ± standard error (*n* = 6). ^1^ CON, piglets fed basal diet; CAT, piglets fed basal diet supplemented with 2.0 g/kg exogenous catalase production. ^2^ CAT, catalase; SOD, superoxide dismutase; GSH-Px, glutathione peroxidase; T-AOC, total antioxidative capacity; MDA, malondialdehyde.

**Table 4:** Effects of dietary exogenous catalase supplementation on jejunum mucosal factors in weaned piglets.

Items ^2^	Treatment ^1^		*p* value
	CON	CAT	
DAO, U/mg protein	8.30 ± 1.10	12.65 ± 0.81	0.010
TGF-α, ng/g protein	16.20 ± 1.31	20.03 ± 0.99	0.042
TFF, ng/g protein	700.55 ± 38.70	753.57 ± 59.82	0.474
MHC-II, ng/mg protein	47.42 ± 2.81	52.04 ± 2.33	0.235

Values are mean ± standard error (*n* = 6). ^1^ CON, piglets fed basal diet; CAT, piglets fed basal diet supplemented with 2.0 g/kg exogenous catalase production. ^2^ DAO, diamine oxidase; TGF-α, transforming growth factor-α; TFF, trefoil factor family; MHC- II, major histocompatibility complex class II.

**Table 5:** Effects of dietary exogenous catalase supplementation on the jejunum mucosal proinflammatory cytokines and SIgA of weaned piglets.

Items ^2^	Treatment ^1^		*p* value
	CON	CAT	
TNF-α, pg/mg protein	34.25 ± 1.83	28.63 ± 1.53	0.040
IL-6, pg/mg protein	41.34 ± 2.19	33.56 ± 2.10	0.028
SIgA, μg/mL	51.26 ± 3.57	66.49 ± 4.58	0.025

Values are mean ± standard error (*n* = 6). ^1^ CON, piglets fed basal diet; CAT, piglets fed basal diet supplemented with 2.0 g/kg exogenous catalase production. ^2^ TNF-α, tumor necrosis factor-α; IL-6, interleukin-6; SIgA, secretory immunoglobulin A.

**Table 6:** Effects of dietary exogenous catalase supplementation on short-chain fatty acids concentration and pH values in the colonic digesta of weaned piglets.

Items	Treatment ^1^		*p* value
	CON	CAT	
pH value	6.04 ± 0.09	5.57 ± 0.10	0.006
Acetate, mmol/g	33.00 ± 2.93	38.24 ± 3.26	0.259
Propionate, mmol/g	11.77 ± 0.90	12.69 ± 0.96	0.501
Butyrate, mmol/g	3.75 ± 0.68	5.40 ± 0.47	0.074
Total SCFAs ^2^, mmol/g	48.51 ± 4.16	56.34 ± 4.42	0.227

Values are mean ± standard error (*n* = 6). ^1^ CON, piglets fed basal diet; CAT, piglets fed basal diet supplemented with 2.0 g/kg exogenous catalase production. ^2^ SCFAs, short-chain fatty acids.

## Supplementary Materials

The following are available online at https://www.mdpi.com/2076-2607/8/3/391/s1, Table S1: Ingredients composition and nutrient levels of basal diets (as-fed basis); Table S2: Effects of dietary exogenous catalase supplementation on the relative abundance of colonic microbiota at the genus level; Table S3: Effects of dietary exogenous catalase supplementation on the colonic microbiota functions.
