# Supplementary material for: Effects of a Diet Supplemented with Exogenous Catalase from Penicillium notatum on Intestinal Development and Microbiota in Weaned Piglets
Source: Microorganisms. 2020 Mar 11;8(3):391. doi: 10.3390/microorganisms8030391 (PMC7143822; doi:10.3390/microorganisms8030391)
Supplement: Supplementary file 1 [file microorganisms-08-00391-s001.pdf]

Table S1. Ingredients composition and nutrient levels of basal diets (as-fed basis).

Items	Phases	
	**1-21 d**	**22-35 d**
Ingredients, %		
Corn	37.55	48.09
Extruded corn	18.00	15.00
Soybean meal	13.00	18.50
Extruded soybean	10.00	6.00
Fish meal	4.00	3.00
Spray-dried plasma protein	3.00	0.00
Whey powder	10.00	5.00
Soy oil	1.03	1.08
Monocalcium phosphate	0.78	0.66
Limestone	0.95	0.90
NaCl	0.30	0.30
L-Lysine HCl	0.32	0.39
DL-Methionine	0.16	0.20
L-Threonine	0.11	0.16
L-Tryptophan	0.00	0.02
Corn starch	0.30	0.20
Vitamin-mineral premix [1]	0.50	0.00
Vitamin-mineral premix [2]	0.00	0.50
Total	100.00	100.00
Nutrient composition [3]		
Digestible energy, Mcal/kg	3.54	3.49
Crude protein, %	20.56	18.88
Ca, %	0.80	0.70
Digestible P, %	0.40	0.34
Lysine, %	1.35	1.24
Methionine, %	0.39	0.36
Threonine, %	0.79	0.73
Tryptophan, %	0.23	0.20

[1] The premix provided for per kg of feed: Zn, 100 mg; Mn, 4 mg; Fe, 100 mg; Cu, 6 mg; I, 0.14 mg; Se, 0.3 mg; choline chloride, 500 mg; vitamin A, 10,500 IU; vitamin D3, 3,300 IU; vitamin E, 22.5 IU; vitamin K3, 3 mg; vitamin B1, 3 mg; vitamin B2, 7.5 mg; vitamin B6, 4.5 mg; vitamin B12, 0.03 mg; niacin, 30 mg; pantothenate, 15 mg; folic acid, 1.5 mg; biotin, 0.12 mg.

[2] The premix provided for per kg of feed: Zn, 80 mg; Mn, 3 mg; Fe,100 mg; Cu, 5 mg; I, 0.14 mg; Se, 0.25 mg; choline chloride, 400 mg; vitamin A, 10,500 IU; vitamin D3, 3,300 IU; vitamin E, 22.5 IU; vitamin K3, 3 mg; vitamin B1, 3 mg; vitamin B2, 7.5 mg; vitamin B6, 4.5 mg; vitamin B12, 0.03 mg; niacin, 30 mg; pantothenate, 15 mg; folic acid, 1.5 mg; biotin, 0.12 mg.

[3] All data were calculated according to the tables of Feed Composition and Nutrient Values in China (2016) in two diets.

Table S2. Effects of dietary exogenous catalase supplementation on the relative abundance of colonic microbiota at the genus level.

Items, %	Treatment [1]		*p* value
	CON	CAT	
Lactobacillus	11.25±2.35	11.96±1.99	0.666
Succinivibrio	2.44±0.68	6.99±2.47	0.069
Alloprevotella	4.90±2.68	5.56±0.98	0.226
Leeia	2.40±0.76	1.75±0.34	0.732
Megasphaera	2.92±0.90	2.91±0.62	0.776
Pseudobutyrivibrio	3.78±1.10	4.13±0.47	0.398
Prevotella_9	2.52±0.91	3.16±0.64	0.286
Streptococcus	2.77±1.03	0.80±0.12	0.021
Prevotellaceae_NK3B31_group	2.37±0.50	1.42±0.25	0.172
Blautia	1.87±0.27	2.19±0.39	0.481
Phascolarctobacterium	2.40±0.85	1.14±0.12	0.250
Lachnospiraceae_XPB1014_group	1.23±0.96	0.52±0.18	0.418
Treponema_2	1.16±0.59	1.30±0.41	0.401
Clostridium_sensu_stricto_1	1.80±0.33	2.66±0.35	0.092
Anaerovibrio	1.47±0.35	1.53±0.33	0.775
Rikenellaceae_RC9_gut_group	2.43±0.62	2.26±0.29	0.829
Faecalibacterium	2.50±0.42	1.77±0.35	0.163
Staphylococcus	0.00±0.00	0.00±0.00	0.363
Escherichia-Shigella	1.76±0.64	0.22±0.02	0.006
Ruminococcaceae_UCG-005	1.33±0.52	0.94±0.14	0.727
Ruminococcaceae_UCG-002	1.66±0.37	1.24±0.15	0.670
Dialister	0.20±0.02	1.04±0.48	0.008
Ruminococcus_1	1.16±0.36	1.49±0.37	0.309
Bifidobacterium	0.02±0.01	0.63±0.54	0.023
Eubacterium_coprostanoligenes_group	1.58±0.44	0.83±0.08	0.178

Values are mean ± standard error (n=6).

[1] CON, piglets fed basal diet; CAT, piglets fed basal diet supplemented with 2.0 g/kg exogenous catalase production.

Table S3. Effects of dietary exogenous catalase supplementation on the colonic microbiota functions.

Items, %	Treatment [1]		*p* value
	CON	CAT	
Nitrate_reduction	1.68±0.29	1.00±0.10	0.054
Nitrogen_respiration	0.94±0.31	0.35±0.07	0.080
Nitrite_respiration	0.93±0.31	0.34±0.07	0.075
Aerobic_chemoheterotrophy	0.99±0.28	0.17±0.02	0.001
Nitrate_respiration	0.90±0.30	0.13±0.01	0.004
Fumarate_respiration	0.88±0.31	0.13±0.01	0.008
Human_pathogens_all	1.14±0.30	0.13±0.01	0.001
Human_pathogens_gastroenteritis	1.09±0.29	0.12±0.01	0.002
Human_pathogens_diarrhea	0.88±0.30	0.12±0.01	0.006

Values are mean ± standard error (n=6).

[1] CON, piglets fed basal diet; CAT, piglets fed basal diet supplemented with 2.0 g/kg exogenous catalase production.
